# Pediatrics ACES and related life event screener (PEARLS): translation, transcultural adaptation, and validation to Brazilian Portuguese

**DOI:** 10.1016/j.jped.2024.10.003

**Published:** 2024-10-29

**Authors:** Luciana Cristina Mancio Balico, Neeta Thakur, Dayna Long, Emerson Rodrigues da Silva, Vandrea Carla de Souza

**Affiliations:** a Universidade de Caxias do Sul, Programa de Pós-Graduação em Ciências da Saúde, Caxias do Sul, RS, Brazil; b University of California San Francisco, Department of Medicine, Division of Pulmonary and Critical Care Medicine, San Francisco, CA, USA; c University of California San Francisco, Benioff Children's Hospital Oakland, Oakland, CA, USA; d Universidade de Caxias do Sul, Curso de Medicina, Caxias do Sul, RS, Brazil

**Keywords:** Adverse childhood experiences, Translation, Validation studies, Screening

## Abstract

**Objective:**

Adverse Childhood Experiences (ACEs) have been associated with negative health outcomes. Screening for ACEs is crucial for improving health results, however, there is a shortage of standardized tools designed for children in Brazilian Portuguese. This study aimed to translate, culturally adapt, and validate the Pediatrics ACES and Related Life Event Screener (PEARLS) for use in Brazil.

**Method:**

The study followed a methodological design for cross-cultural adaptation and psychometric evaluation. The PEARLS was translated and culturally adapted following a methodology that includes translation, synthesis, expert committee evaluation, target audience evaluation, and back-translation. After adaptation, a pilot cross-sectional study was conducted at a Multidisciplinary Health Care Clinical Center and a General Hospital-Reference Center for Child and Adolescent Care to assess the instrument's internal consistency, convergent validity, content validity and test-retest reliability.

**Results:**

The PEARLS-Br versions for Children, Teens, and Teen Self-Report were developed and subjected to pilot testing with 202 subjects. Participants demonstrated excellent comprehension, with Verbal Rating Scale median scores of 4 (IQR 4–5). Internal consistency was high, with Cronbach's alpha coefficients ranging from 0.78 to 0.81. Content validity, assessed by Kappa, indicated slight to almost perfect agreement across constructs. Test-retest reliability, assessed by Spearman's correlation coefficient, ranged from 0.89 to 0.94.

**Conclusions:**

PEARLS-BR (Child, teen, and teen self-report versions) were successfully translated, culturally adapted, and validated for assessing ACEs in Brazilian children and adolescents. This tool fills a crucial gap in ACE assessment in the Brazilian context, aligning with global recommendations for screening ACEs to improve overall health outcomes.

## Introduction

Adverse Childhood Experiences (ACEs) are defined as stressful or traumatic events that children experience before the age 18 years.^1^ These adversities are associated with the development of long-term health conditions.^2^, ^3^, ^4^ The landmark ACE Study conducted in adults revealed a clear dose-response relationship between ACE scores and their impact on health, indicating that ACE scores are associated with an increased likelihood of developing severe psychological issues such as substance dependency, depression, and suicide, as well as physical conditions including hepatitis, cardiac ischemia, lung cancer, and chronic obstructive pulmonary disease in adulthood.^2^

The ACE Study led to the development of the “Pediatrics ACEs and Related Life Event Screener” (PEARLS), facilitating early intervention. Recent studies have linked ACEs in children to compromised executive functioning,^3^^,^^5^ respiratory issues,^3^ immune/endocrine harm,^3^^,^^4^ asthma,^3^^,^^6^ infections,^3^^,^^6^ and learning difficulties.^3^^,^^7^ In adolescents, risks like obesity,^8^ violence,^9^ and smoking.^10^ These findings underscore the importance of early identification and intervention.

The link between ACEs and poor health may result from abnormal HPA axis functioning, assessed through cortisol and C-reactive protein (CRP).^11^ This chronic stress response leads to heightened inflammation, increasing susceptibility to infections and autoimmune conditions.^12^ Inflammatory and epigenetic markers, such as C-reactive protein and telomeres,^13^ are implicated in the long-lasting impact of ACEs, potentially influencing intergenerational transmission. Timely identification of ACEs is crucial to break the cycle of toxic stress.

While screening for ACEs is recommended by prominent health organizations, including the Center for Disease Control and Prevention (CDC),^14^ the American Academy of Pediatrics (AAP),^4^ and the World Health Organization (WHO),^1^ there is currently a gap in research regarding prevalence and associations of ACEs with physical and psychological diseases in Brazilian children. Additionally, the absence of validated and standardized instruments in Brazilian Portuguese for ACE assessment highlights the need for validated tools that are culturally and contextually appropriate. The use of non-validated instruments can result in inaccurate assessments, leading to inappropriate interventions, which ultimately compromise the quality of care.

Given the vulnerability of the developing brain to adversities, early detection of ACEs is essential to prevent chronic toxic stress. Therefore, this pioneering study, the first outside the United States, aims to translate, culturally adapt, and validate the PEARLS questionnaire through the process of reliability testing, face validity, and gathering evidence based on content and internal structure. The goal is to establish the PEARLS-BR as a reliable tool for use in Brazilian pediatric practice, supporting the early identification of ACE-related diseases.

## Materials and methods

The cultural adaptation of the PEARLS (Child, Teen, and Teen self-report versions) was conducted with authorization from the developers under a licensing agreement signed between the University of California San Francisco (UCSF), the copyright holder of the instrument, and the Universidade de Caxias do Sul (UCS). This research received approval from the UCS Research Ethics Committee (number 6.090.525).

### Instruments

#### PEARLS

PEARLS is a self-applicable questionnaire designed to screen for ACEs in individuals from birth to 18 years old. The PEARLS Child and Teen versions have 17 questions, with two additional ones in the Teen version. The items assess sexual, physical, or emotional violence; neglect; parental mental illness, substance abuse, incarceration; separation/divorce; domestic violence, as well as food insecurity, housing instability, community violence, and discrimination. PEARLS Child and Teen are completed by a caregiver, while the PEARLS Teen Self-Report is completed by the adolescent. Scoring is based on the sum of affirmative responses, which can be interpreted either continuously - assessing ACEs, Related Life Events, or the total score for a detailed view of experiences - or categorized into groups (0, 1–3, 4+) to identify different levels of exposure.^3^^,^^15^

PEARLS was developed and validated in the USA, showing good psychometric properties with reliability (α = 0.61–0.87), factor loadings between 0.44 and 0.83, and a three-factor model (Maltreatment, Household Challenges, Social Context) confirmed via CFA with satisfactory fit indices (Χ²(116) = 139.68, *p* = .07; RMSEA = 0.03; CFI = 0.99; TLI = 0.99).^4^

#### QUESI

The Childhood Trauma Questionnaire (CTQ), validated for the Brazilian population as the “*Questionário sobre Traumas na Infância*” (QUESI), is designed for adolescents (12+) and adults to rate the frequency of 28 childhood trauma experiences on a five-point Likert scale. It assesses sexual, physical, or emotional abuse, as well as emotional and physical neglect. Validation studies in Brazil, including CFA, confirmed appropriate factor structure (χ²(270) = 1174.22, *p* < .0001; CFI = 0.98; TLI = 0.98; RMSEA = 0.04) and internal consistency (α = 0.80 to 0.91). In this study, QUESI was used to assess the convergent validity of the PEARL-BR, completed by caregivers and teenagers (13+).

### Procedure

**Figure 1:**
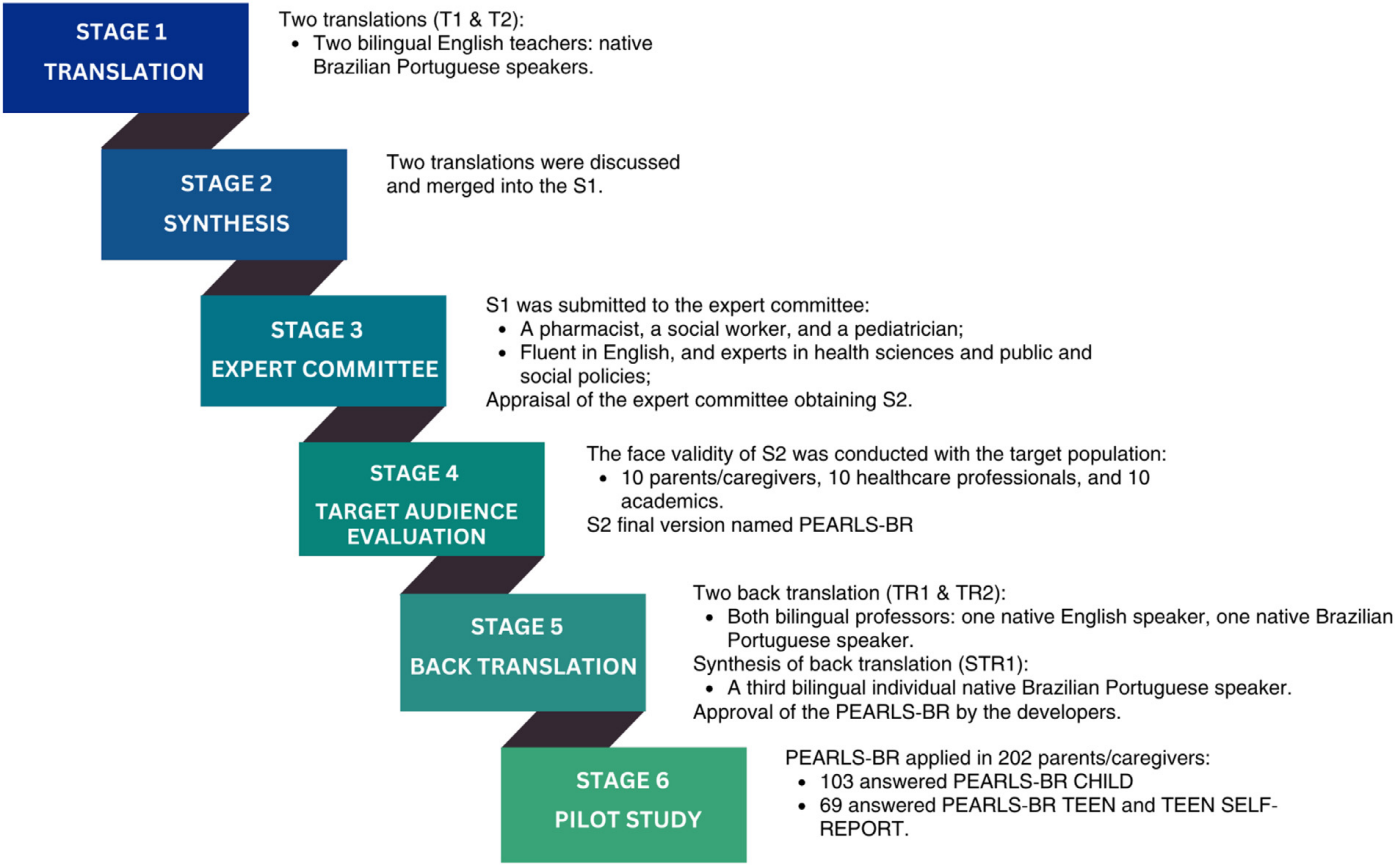
The graphical representation of stages for transcultural adaptation of PEARLS, adapted from Borsa et al.^16^^,a^ ^a^Modified drawing from Pereira et al.^27^

The study followed Borsa et al.'s six-stage model for cross-cultural adaptation and psychometric evaluation (Figure 1).^16^ After the adaptation, a pilot cross-sectional study was conducted to evaluate the psychometric properties of the instrument.

### Evidence-based on content

The PEARLS was translated from English to Brazilian Portuguese by two bilingual professors, whose native language is Portuguese, resulting in two translated versions (T1 and T2) that were merged into Synthesis 1 (S1).

The S1 was submitted to the appraisal of an expert committee composed of a pharmacist, a social worker, and a pediatrician, selected based on their academic background, professional experience in health sciences, public policies, and social policies, as well as their proficiency in English. These experts were chosen for their relevant expertise in these fields, holding doctoral degrees, actively conducting research, and serving as permanent faculty members at a University in Brazil. Each specialist received individual instructions outlining the evaluation task (Supplementary Material 1) The content validity was analyzed by the experts, considering the four dimensions suggested by the literature: semantic, idiomatic, experiential, and conceptual.^17^^,^^18^ Additionally, aspects such as structure, layout, instrument instructions, comprehensiveness, and adequacy of expressions in each item were also evaluated.^16^ They also compared the original instrument and the S1, ensuring the best fit with the context and experiences of Brazilian children and teens. Each expert submitted their evaluations with comments to the responsible researchers, and these evaluations were identified as J1, J2, and J3 (Supplementary Material 2). The committee collectively discussed each suggestion for each item, arriving at a conclusion together, successfully consolidating and obtaining synthesis 2 (S2).

The face validity was conducted with the target population to assess content validation and the overall meaning of S2. The instrument was evaluated not only by a convenience sample of individuals treated at a Multidisciplinary Health Care Center but also by healthcare professionals (nutritionist, pediatrician, pediatric resident, nephrology resident, pediatric neurologist, physical therapist, social worker, nurse technician, psychologist, and nurse) and academics from health sciences fields (medicine, nutrition, and physiotherapy). Including these professionals was essential as their insights provided valuable perspectives on the instrument's applicability and relevance within a clinical setting. This approach mirrors the methodology of the original PEARLS validation, which also included input from clinic providers and staff to ensure a breadth of response. A total of 30 participants were individually interviewed after signing the informed consent form. They evaluated the clarity, comprehensibility, and adequacy of S2 using a structured feedback form (Supplementary Material 3). This form allowed participants to assess each item of the instrument and suggest modifications, including potential synonyms or changes in wording to enhance the instrument's clarity and relevance. Based on the received suggestions and the analysis of the interviewees’ feedback (Supplementary Material 4), the expert committee decided to maintain version S2 without significant alterations.

The version S2 of the instrument was translated back into English by two different translators. One translator is a bilingual professor whose native language is English (TR1), while the other is a bilingual professor whose native language is Portuguese (TR2). This stage aimed to assess how well the translated version (S2) reflected the content of the original version. Two back translations of the instrument (TR1 and TR2) were obtained, and a third bilingual individual synthesized them into a document named S1TR. Thus, PEARLS-BR was obtained and had the approval of the developer.

### Evidence-based on relationships with external variables

The PEARL-BR pilot study took place from June 2023 to January 2024 at a Multidisciplinary Health Care Center and a General Hospital-Reference Center for Child and Adolescent Care. The General Hospital, part of the 5th Regional Health Coordination of Rio Grande do Sul, covers 49 cities and approximately 1.2 million people. Families were recruited from a convenience sample in the waiting room, where caregivers signed consent forms and adolescents signed assent forms. The goal was to recruit 200 caregivers, who had to meet the following criteria: aged over 18, primary caregiver of a child aged 18 or younger, Brazilian Portuguese speaker, and not have another child in the study.

During the pilot study phase, participants completed the PEARL-BR questionnaire, and the QUESI, and answered sociodemographic information. Specifically, parents of children aged 0 to 12 years responded to the PEARLS-BR Child version, while parents of children aged 13 to 18 years completed the PEARLS-BR Adolescent version. Adolescents aged 13 to 18 years answered the PEARLS-BR Adolescent Self-Report version. The QUESI was completed by all parents and by adolescents aged 13 years and older. Only the parents provided sociodemographic information. Following this, they responded to a five-point verbal rating scale (VRS) aimed at assessing the overall clarity of the instrument, with both parents and adolescents aged 13 and older completing this rating. The pivotal query of this scale was: "Did you understand what was asked in the PEARLS-BR questionnaire?" Scores ranged from a minimum of "1″ ("no comprehension") to a maximum of "5″ ("complete comprehension"). A score of three or below denoted inadequate understanding. Moreover, alongside caregivers, 10 healthcare professionals recruited by a convenience sample from the Multidisciplinary Health Care Center also provided feedback through the VRS after signing the informed consent form. The test-retest reliability assessment, a key measure of reliability, was conducted 30 days after caregivers completed the PEARLS-BR in the pilot study, with the aim of obtaining a minimum of 50 retests.

### Statistical analysis

All study data were collected and managed using REDCap (Research Electronic Data Capture), hosted at UCS.^19^^,^^20^ REDCap is a secure platform for research data capture. Statistical analysis was conducted using R software (version 3.5.2). Continuous variables were expressed as median and interquartile range. Internal consistency was assessed with Cronbach's alpha, where values above 0.75 indicated satisfactory consistency. The 95 % confidence intervals for Cronbach's alpha were computed using the Duhachek method (PSYCH package). Comprehension and semantic equivalence were evaluated with the VRS, with scores above three indicating adequate understanding. Kappa coefficients assessed content validity, with values over 0.60 considered satisfactory. Test-retest reliability was determined using Spearman's correlation, with values above 0.75 indicating reliable consistency.

## Results

The stages and procedures for translation, adaptation, and validation followed the flow outlined in Figure 1. Supplementary Material 5 presents the instrument's items in their original versions, progressing through each phase until the final version.

To enhance clarity in the title, the term “adverse childhood experiences” was chosen instead of the acronym “ACE's”. In the original PEARLS, questions were in the present perfect tense, but since this tense does not exist in Brazilian Portuguese, the past perfect indicative was used to indicate that any past action should be considered positive in the response.

The expert committee raised eight points: seven on equivalence and one on layout. Four of these were modified resulting in S2 (final version), including layout adjustments with increased spacing while keeping the original PEARLS structure. Additionally, question 6 of part 1 was revised for semantic equivalence, and question 2 was expanded to include examples of mental illnesses. Question 9 of part 2 was also revised to align with the original text. The other four points remained unchanged after analysis.

During the evaluation phase, 30 participants, including healthcare professionals, caregivers, and academics, reviewed each component of PEARLS-BR. Of these, 80 % (*n* = 24) found the version clear and suitable for the Brazilian context, while 20 % (*n* = 6) provided feedback leading to an item-by-item review. After consultations with the expert committee, version S2 was maintained without changes. The original PEARLS authors approved the PEARLS-BR version after considering nuances in Brazilian grammar and gender-specific endings.

To ensure the validity of the study, a pilot study was conducted involving the preliminary application of the instrument in a sample representing the target population. A total of 202 caregivers participated: 133 responded to the PEARLS-BR Child, while 69 completed the PEARLS-BR Teen, and 69 adolescents over 13 years old completed the PEARLS-BR Teen Self-Report. Throughout this process, the appropriateness of the items in terms of their meaning and comprehensibility, as well as the instructions for test administration, was assessed. No adjustments were deemed necessary, indicating the readiness of the instrument for use. Table 1 presents the sociodemographic characteristics of the caregivers who completed the PEARLS-BR Child version, along with the characteristics of their children (ages 0–12), as well as the caregivers who completed the PEARLS-BR Teen version. Additionally, it includes the sociodemographic characteristics of the adolescents (ages 13–18) who completed the PEARLS-BR Teen Self-Report independently. This structure clarifies the distinct groups: caregivers reporting on their children, caregivers reporting on their teens, and the adolescents themselves providing self-reports.

**Table 1:** Sociodemographic characteristics of caregivers and their children (PEARLS-BR Child) and caregivers and their adolescents (PEARLS-BR teen and teen self-report).

Characteristics	PEARLS-BR CHILD version (*n* = 133)		PEARLS-BR TEEN version (*n* = 69)	PEARLS-BR TEEN SELF-REPORT version (*n* = 69)
	Caregivers	Children	Caregivers	Teens
Age, median (IQR)	36 (28–42)	7 (4–10)	40 (34–47)	14 (13–14)
Sex, n (%)				
Male	27 (20.3)	54 (40.6)	6 (8.7)	13 (18.8)
Female	106 (79.7)	79 (59.4)	63 (91.3)	56 (81.2)
Ethnic-racial classification, n (%)				
White	82 (61.7)	88 (66.2)	41 (59.4)	46 (66.7)
Black	6 (4.6)	3 (2.3)	5 (7.2)	6 (8.7)
Brown/Mixed-race	42 (31.5)	40 (30.1)	22 (31.9)	16 (23.1)
Indigenous	3 (2.2)	1 (0.7)	1 (1.5)	0
Asian	0	1 (0.7)	0	1 (1.5)
undergraduate Degree				
	0	20 (15.0)	0	0
	1 (0.7)	34 (25.6)	0	0
	7 (5.3)	58 (43.6)	15 (21.7)	1 (1.5)
	31 (23.3)	21 (15.8)	19 (27.5)	46 (66.7)
	81 (60.9)	0	32 (46.4)	21 (30.3)
	11 (8.3)	0	3 (4.4)	1 (1.5)
	2 (1.5)	0	0	0
Caregiver-child relationship, n (%)				
Father	23 (17.3)		5 (7.2)	
Mother	95 (71.4)		51 (73.9)	
Grandfather	3 (2.2)		1 (1.5)	
Grandmother	5 (3.8)		9 (13.0)	
Aunt	5 (3.8)		1 (1.5)	
Others	2 (1.5)		2 (2.9)	
Marital Status, n (%)				
Single	51 (38.4)		26 (37.7)	
Married	42 (31.5)		19 (27.5)	
Civil Union	21 (15.8)		15 (21.7)	
Separated	15 (11.3)		5 (7.2)	
Widowed	4 (3.0)		4 (5.9)	
Income (in R$/per month)				
2589.00 or less	76 (57.1)		41 (59.4)	
Greater than 2589.00	57 (42.9)		28 (40.6)	
Housing Type, n (%)				
House	112 (84.2)		65 (94.1)	
Apartment	19 (14.3)		4 (5.9)	
Shared housing	2 (1.5)		0	
Number of rooms, median (IQR)	5 (4–6)		6 (5–7)	
Number of cohabitants, median (IQR)	4 (3–5)		4 (3–5)	

The VRS within the PEARLS-BR Child and Teen, as evaluated by caregivers, demonstrated strong comprehension and semantic equivalence, achieving a median score of 4 (IQR: 4–5). Similarly, adolescents completing the PEARLS-BR Teen Self-Report rated the instrument similarly, with a median score of 4 (IQR: 4–5). Additionally, ten healthcare professionals assessed comprehension and semantic equivalence, yielding a median score of 5 (IQR: 4–5).

Measurement of internal consistency, as assessed through Cronbach's alpha, revealed high reliability across all versions of PEARLS-BR. Specifically, the Child version exhibited an alpha coefficient of 0.81 (95 % CI: 0.77–0.86), the Teen version 0.79 (95 % CI: 0.72–0.85), and the Teen Self-Report version 0.78 (95 % CI: 0.71–0.85).

At the outset of this analysis, it is pertinent to clarify that the levels of agreement are being assessed in accordance with the QUESI. The convergent validity, assessed using Kappa, indicated varying degrees of agreement for the emotional abuse construct across different versions of the instrument: moderate agreement (0.50) for the Child version, fair agreement (0.33) for the Teen version, and moderate agreement (0.51) for the Teen Self-Report version. Similarly, moderate agreement was observed for the physical abuse construct across all versions, with values of 0.52, 0.47, and 0.49 for the Child, Teen, and Teen Self-Report versions, respectively. Regarding the sexual abuse construct, substantial agreement (0.67, 0.79) and almost perfect agreement (0.83) were noted across the same versions. Furthermore, the physical neglect construct showed slight agreement (0.17, 0.15) for the Child and Teen versions, and fair agreement (0.26) for the Teen Self-Report version. The instrument used to make these comparisons measures slightly different outcomes, thereby providing additional context for interpreting the agreement levels.

The test-retest reliability results from the Spearman test demonstrated a strong positive and statistically significant agreement between the initial and subsequent responses, obtained after a 30-day interval. Specifically, for the PEARLS-BR Child version, ρ = 0.91; for the PEARLS-BR Teen version, ρ = 0.90; and for the PEARLS-BR Teen self-report version, ρ = 0.94. In this phase, a total of 61 participants were involved, with 43 responding to the Child version and 18 to the Teen and Teen Self-Report versions, respectively.

The final version of PEARL-BR Child is available in Supplementary Material 6, while PEARLS-BR Teen can be found in Supplementary Material 7, and PEARLS-BR Teen Self-Report is available in Supplementary Material 8.

## Discussion

This study presents the Brazilian Portuguese version of PEARLS, tailored for screening ACEs from birth to eighteen years in Brazil, following a rigorous process of translation, cultural adaptation, and validation.^17^^,^^18^ The methodology adhered to guidelines proposed by Borsa et al.,^16^ previously successful in Brazil for translating and validating several instruments.^21^, ^22^, ^23^

The PEARLS-BR addresses a critical gap in screening ACEs within the Brazilian population, focusing specifically on individuals aged from birth to eighteen years old, unlike other tools limited to those above 12 years. The involvement of caregivers and healthcare professionals was crucial for refining the screener. Engaging with diverse stakeholders ensured semantic, idiomatic, and conceptual equivalence, making the tool comprehensible and applicable to the target population. The VRS, which measured comprehension and semantic equivalence, was also adequate for the tool's face validity.

The PEARLS-BR demonstrated satisfactory internal consistency and strong test-retest reliability across all three versions, indicating robust psychometric properties. These results are consistent with the psychometric analyses of the original PEARLS, which also showed high internal consistency and reliability.^1^^,^^4^ The PEARLS-BR effectively identified traumatic experiences during childhood and adolescence, particularly in cases of sexual abuse in the Teen Self-Report version.

QUESI was used in the convergent validity analysis, focusing on specific types of maltreatment,^24^^,^^25^ while PEARLS assesses a broader range of ACEs, including household challenges.^1^^,^^4^ The findings indicate moderate to substantial agreement between the two instruments, particularly regarding sexual and physical abuse, reinforcing the convergent validity of the PEARLS-BR. These results align with the psychometric performance of PEARLS in the USA, confirming its utility for screening ACEs in Brazilian children and adolescents.

Despite data collection being limited to southern Brazil, the PEARLS-BR can be used nationwide due to its rigorous validation process, consistent with other Brazilian scales.^16^^,^^21^, ^22^, ^23^ The tool's reliability and applicability in diverse healthcare settings facilitate open communication and comfortable disclosure of sensitive information.

The PEARLS-BR serves as a practical tool for healthcare professionals in primary care settings, aiming to screen ACEs for children and adolescents. This instrument, similar to the original PEARLS, is not intended for psychodiagnostics but rather as a valuable resource for professionals who may lack access to specialized psychology or social services.^1^ It also serves as a valuable research instrument, facilitating studies aimed at understanding and addressing ACEs in diverse populations.

Further research is needed to develop treatments and interventions for ACEs. Routine screening can provide crucial data to assess the economic burden of ACEs,^26^ and guide investment in strategies to improve population health. However, the limitations of this study were the absence of confirmatory factor analysis (CFA) and measurement invariance testing, which limits the ability to fully validate the factor structure of the PEARLS-BR across different subgroups, such as age or gender. Future studies should focus on conducting CFA and assessing measurement invariance across diverse populations to validate construct validity.

Pearls-BR underwent a successful translation into Brazilian Portuguese, along with cultural adaptation and validation, enabling its utilization for assessing ACEs within the pediatric Brazilian population.

## Funding sources

L.B. was supported by CAPES: "This study was financed in part by the Coordenação de Aperfeiçoamento de Pessoal de Nível Superior - Brasil (CAPES) - Finance Code 001″.

## Conflicts of interest

The authors declare no conflicts of interest.

## Appendix Supplementary materials

Image, application 1

Image, application 2

Image, application 3

Image, application 4

Image, application 5

Image, application 6

Image, application 7

Image, application 8

## Footnotes

Supplementary material associated with this article can be found in the online version at doi:10.1016/j.jped.2024.10.003.

## References

[1] Koita K., Long D., Hessler D., Benson M., Daley K., Bucci M. (2018). Development and implementation of a pediatric adverse childhood experiences (ACEs) and other determinants of health questionnaire in the pediatric medical home: a pilot study. PLoS ONE.

[2] Felitti V.J., Anda R.F., Nordenberg D., Williamson D.F., Spitz A.M., Edwards V. (1998). Relationship of childhood abuse and household dysfunction to many of the leading causes of death in adults. The Adverse Childhood Experiences (ACE) study. Am J Prev Med.

[3] Thakur N., Hessler D., Koita K., Ye M., Benson M., Gilgoff R. (2020). Pediatrics adverse childhood experiences and related life events screener (PEARLS) and health in a safety-net practice. Child Abuse Negl.

[4] Ye M., Hessler D., Ford D., Benson M., Koita K., Bucci M. (2023). Pediatric ACEs and related life event screener (PEARLS) latent domains and child health in a safety-net primary care practice. BMC Pediatr.

[5] Bucci M., Marques S.S., Oh D., Harris N.B. (2016). Toxic stress in children and adolescents. Adv Pediatr.

[6] Wing R., Gjelsvik A., Nocera M., McQuaid E.L. (2015). Association between adverse childhood experiences in the home and pediatric asthma. Ann Allergy Asthma Immunol.

[7] Bethell C.D., Newacheck P., Hawes E., Halfon N. (2014). Adverse childhood experiences: assessing the impact on health and school engagement and the mitigating role of resilience. Health Aff (Millwood).

[8] Zhang Y., Li Y., Jiang T., Zhang Q. (2023). Role of body mass index in the relationship between adverse childhood experiences, resilience, and mental health: a multivariate analysis. BMC Psychiatry.

[9] Forster M., Gower A.L., McMorris B.J., Borowsky I.W. (2017). Adverse childhood experiences and school-based victimization and perpetration. J Interpers Violence.

[10] Parks M.J., Davis L., Kingsbury J.H., Shlafer R.J. (2020). Adverse childhood experiences and youth cigarette use in 2013 and 2016: emerging disparities in the context of declining smoking rates. Nicotine Tob Res.

[11] Dempster K.S., O'Leary D.D., MacNeil A.J., Hodges G.J., Wade T.J (2021). Linking the hemodynamic consequences of adverse childhood experiences to an altered HPA axis and acute stress response. Brain Behav Immun.

[12] De La Rosa R., Zablotny D., Ye M., Bush N.R., Hessler D., Koita K. (2023). Biological burden of adverse childhood experiences in children. Psychosom Med.

[13] Lang J., McKie J., Smith H., McLaughlin A., Gillberg C., Shiels P.G. (2020). Adverse childhood experiences, epigenetics and telomere length variation in childhood and beyond: a systematic review of the literature. Eur Child Adolesc Psychiatry.

[14] Preventing adverse childhood experiences (ACEs): leveraging the best available evidence; 2019. [Accessed Feb 25, 2024]. Available from: https://stacks.cdc.gov/view/cdc/82316

[15] Koita K., Long D., Hessler D., Benson M., Daley K., Bucci M. (2018). Development and implementation of a pediatric adverse childhood experiences (ACEs) and other determinants of health questionnaire in the pediatric medical home: a pilot study. PLoS ONE.

[16] Borsa J.C., Damásio B.F., Bandeira D.R. (2012). Cross-cultural adaptation and validation of psychological instruments: some considerations. Paidéia (Ribeirão Preto).

[17] Beaton D.E., Bombardier C., Guillemin F., Ferraz M.B. (2000). Guidelines for the process of cross-cultural adaptation of self-report measures. Spine (Phila Pa 1976).

[18] Guillemin F., Bombardier C., Beaton D. (1993). Cross-cultural adaptation of health-related quality of life measures: literature review and proposed guidelines. J Clin Epidemiol.

[19] Harris P.A., Taylor R., Minor B.L., Elliott V., Fernandez M., O'Neal L. (2019). The REDCap consortium: building an international community of software platform partners. J Biomed Inform.

[20] Harris P.A., Taylor R., Thielke R., Payne J., Gonzalez N., Conde J.G. (2009). Research electronic data capture (REDCap)–a metadata-driven methodology and workflow process for providing translational research informatics support. J Biomed Inform.

[21] Borsa J.C., Bandeira D.R. (2014). [Cross-cultural adaptation of peer aggressive and reactive behaviors questionnaire in Brazil]. Psico-USF.

[22] Patias N.D., Machado W.D., Bandeira D.R., Dell'Aglio D.D (2016). [Depression anxiety and stress scale (DASS-21) - short form: adaptation and validation for Brazilian adolescents]. Psico-USF.

[23] Donat J.C., Lobo N dos S., Jacobsen G dos S., Guimarães E.R., Kristensen C.H., Berger W. (2019). Translation and cross-cultural adaptation of the international trauma questionnaire for use in Brazilian Portuguese. Sao Paulo Med J.

[24] Grassi-Oliveira R., Stein L.M., Pezzi J.C. (2006). [Translation and content validation of the childhood trauma questionnaire into Portuguese language]. Rev Saude Publica.

[25] Grassi-Oliveira R., Cogo-Moreira H., Salum G.A., Brietzke E., Viola T.W., Manfro G.G. (2014). Childhood trauma questionnaire (CTQ) in Brazilian samples of different age groups: findings from confirmatory factor analysis. PLoS ONE.

[26] Peterson C., Aslam M.V., Niolon P.H., Bacon S., Bellis M.A., Mercy J.A. (2023). Economic burden of health conditions associated with adverse childhood experiences among US adults. JAMA Netw Open.

[27] Pereira R.P., Leitão A.Q., Fotakos G.S., Neves dos Reis J., Rocha F.E., Machado M.G. (2023). Pediatric incontinence questionnaire (PINQ): translation and transcultural adaptation to Brazilian Portuguese. J Pediatr (Rio J).

