# Supplementary material for: Pediatrics ACES and related life event screener (PEARLS): translation, transcultural adaptation, and validation to Brazilian Portuguese
Source: J Pediatr (Rio J). 2024 Oct 29;101(2):262–8. doi: 10.1016/j.jped.2024.10.003 (PMC11889689; doi:10.1016/j.jped.2024.10.003)
Supplement: Supplementary file 1 [file mmc1.pdf]

EVALUATION BY THE EXPERT COMMITTEE – PEARLS PART 3:

You are invited to join the expert panel to evaluate the translation, adaptation, and synthesis of the PEARLS instrument (Pediatric ACEs and Related Life Events Screener).

We ask that you review the two translations, carried out by two professors from the *UCS Writing Center*, and the synthesis of these translations, conducted by PhD felllow Luciana Balico in collaboration with the two professors, comparing them with the original instrument.

At this time, please evaluate only the aspects described in the table below, indicating whether the item is met or not. If you wish, you may add comment boxes for further discussion:

Evaluated Item	Met	
	Yes	No
Were two translations presented by independent bilingual translators?		
In the presented synthesis, is there semantic equivalence? (Do the words convey the same meaning as the original, and are there no grammatical errors in the translation?)		
In the presented synthesis, is there idiomatic equivalence? (Evaluate whether the items that were difficult to translate from the original instrument have been adapted with an equivalent expression that has not altered the cultural meaning of the item.)		
In the presented synthesis, is there experiential equivalence? (Determine whether a specific item from the original instrument is applicable in the new culture, and if not, substitute it with an equivalent item.)		
In the presented synthesis, is there conceptual equivalence? (Evaluate whether a particular term or expression, even if translated correctly, assesses the same aspect in Brazil.)		
Are the sentences understandable and consistent with the fluency of the target language?		
Is the structure, layout, and formatting of the instrument appropriate? (Include in the analysis the font type, font size, and arrangement of information on the instrument.)		
Are the instructions of the instrument adequate (clarity of the rapport)?		
Is the instrument appropriate for its intended scope (Brazilian families)?		

Can the terms and expressions contained in the instrument's questions be generalized to different contexts and populations (i.e., different regions of Brazil)?		
After the discussion with the expert panel members, were all versions of the instrument consolidated, achieving consensus on the pre-final version for pre-testing (evaluation with a small sample of the target audience)?		

( ) Approved; or

( ) Translation must be repeated; or

( ) The unmet items listed above must be corrected.

EVALUATOR COMMENTS:

Name/Signature:

Date:
