# Supplementary material for: Pediatrics ACES and related life event screener (PEARLS): translation, transcultural adaptation, and validation to Brazilian Portuguese
Source: J Pediatr (Rio J). 2024 Oct 29;101(2):262–8. doi: 10.1016/j.jped.2024.10.003 (PMC11889689; doi:10.1016/j.jped.2024.10.003)
Supplement: Supplementary file 2 [file mmc2.pdf]

Suggested Changes by the Expert Judges Committee that Resulted in S2.

Item	Original	S1	Comments/Suggestions from the Expert Judges	Justification	S2
Layout	N/A	N/A	Information presented together without visual distinction.	Layout modified with increased spacing between lines.	N/A
Page 1 Item 4	Has the child's biological parent or any caregiver ever had, or currently has a problem with too much alcohol, street drugs or prescription medications use	Um dos pais biológicos da criança ou algum responsável já teve, ou atualmente tem um problema com o uso excessivo de álcool, drogas ou medicamentos prescritos?	J1: "*street drugs"* was translated as "drugs." There is a difference between street drugs and other drugs.	The translation for "drugs" was maintained, understanding that the use of any substances that alter the parents' behavior should be considered by the respondent.	Um dos pais biológicos da criança ou algum responsável já teve, ou atualmente tem um problema com o uso excessivo de álcool, drogas ou medicamentos prescritos?
Page 1 Item 2	Has your child ever lived with a parent/caregiver who had mental health issues? (for example, depression, schizophrenia, bipolar disorder, PTSD, or an anxiety disorder)	Seu filho(a) já morou com um dos pais/ responsável que apresentava problemas de saúde mental?	J3: To maintain correspondence with the original questionnaire, examples of illnesses (for example, depression, schizophrenia, bipolar disorder, PTSD, or an anxiety disorder) should be included.	Examples of mental illnesses have been added as per the original.	Seu filho(a) já morou com um dos pais/responsável que apresentava problemas de saúde mental? (Por exemplo, depressão, esquizofrenia, transtorno bipolar, transtorno de estresse pós-traumático e/ou transtorno de ansiedade.)

Page 1<br>Item 6	Has your child seen or heard a parent/caregiver:<br>*(Mark yes, if any are true for you or your family)*<br>screamed at, sworn at, insulted, or humiliated by someone they know?<br>ever slapped, kicked, punched, beaten up or hurt with a weapon?	Seu filho já viu ou ouviu um dos pais/responsável: (assinale sim, se qualquer um for verdadeiro para você ou sua família).<br><br>sendo gritado(a), xingado(a), insultado(a) ou humilhado(a) por outro adulto?<br>sendo esbofeteado(a), chutado(a), socado(a), espancado(a) ou ferido(a) com uma arma?	J3: The term "*sendo gritado*" does not maintain the same equivalence in Portuguese. It needs revision.	In discussion with the committee, it was decided to replace the term "sendo gritado" with "sendo tratado a gritos ou xingado...". This way, the equivalence in Portuguese is maintained, making it easier for the population to understand.	Seu filho(a) já viu ou ouviu um dos pais/responsável: (Assinale sim, se qualquer um for verdadeiro para você ou sua família).<br>Sendo tratado a gritos ou xingado(a), insultado(a) ou humilhado(a) por outro adulto?<br>OU sendo esbofeteado(a), chutado(a), socado(a), espancado(a) ou ferido(a) com uma arma?
Page 2<br>Item 1	Has your child ever seen, heard, or been a victim of violence in your neighborhood, community, or school?<br>*(For example, targeted bullying, assault, or other violent actions, war or terrorism)*	Seu filho(a) já viu, ouviu ou foi vítima de violência em seu bairro, comunidade ou escola? (por exemplo, bullying direcionado, agressão ou outras ações violentas, guerra ou terrorismo)	J3: n Brazil, the terms "*Guerra e terrorismo*" are not part of everyday life. Violence in general is often associated with robberies, executions, shootings, and drug trafficker wars, etc.	The conception and perception of "*war, terrorism, and violence*" are individual to the reader. Other Portuguese-speaking countries may use this instrument; therefore, it was decided to retain the terms "*guerra e terrorismo.*"	Seu filho(a) já viu, ouviu ou foi vítima de violência em seu bairro, comunidade ou escola? (Por exemplo, bullying direcionado, agressão ou outras ações violentas, guerra ou terrorismo).

Page 2<br>Item 2	Has your child experienced discrimination? (for example, being hassled or made to feel inferior or excluded because of their race, ethnicity, gender identity, sexual orientation, religion, learning differences, or disabilities).	Seu filho(a) já experienciou discriminação?<br>(por exemplo, foi incomodado ou se sentiu inferior ou excluído devido à sua raça, etnia, identidade de gênero, orientação sexual, religião, dificuldades de aprendizagem ou deficiências).	J3: Review the term that best represents the word “raça” in the context of the Portuguese language.	The term used by IBGE for the biological characteristic that best represents skin color phenotype is the word “raça.” Therefore, the word “raça” was retained.	Seu filho(a) já experienciou discriminação?<br>(Por exemplo, foi incomodado ou se sentiu inferior ou excluído devido à sua raça, etnia, identidade de gênero, orientação sexual, religião, dificuldades de aprendizagem ou deficiências.)
Page 2<br>Item 3	Has your child ever had problems with housing?<br>*(For example, being homeless, not having a stable place to live, moved more than two times in a six-month period, faced eviction or foreclosure, or had to live with multiple families or family members).*	Seu filho(a) já teve problemas de moradia?<br>(por exemplo, ser sem-teto, não ter um lugar estável para morar, se mudar mais de duas vezes em um período de seis meses, enfrentou despejo ou execução hipotecária ou teve que viver com várias famílias ou membros da família)	J3: The term “*morador de rua*” may be more understandable and adequately represent the original “homeless” than the term “*sem-teto.”*	“*Morador de rua*” is very specific. It is also considered a “*sem-teto*,” but it refers to an extreme situation. The term “*sem-teto*” is used for people who do not have a home but have found an alternative, such as living in a shack, in an occupation, or at someone else's house. “*Sem-teto*” encompasses a much larger population than “*morador de rua*.” Therefore, the term “*sem-teto*” will be retained.	Seu filho(a) já teve problemas de moradia?<br>(Por exemplo, ser sem-teto, não ter um lugar estável para morar, se mudar mais de duas vezes em um período de seis meses, enfrentou despejo ou execução hipotecária ou teve que viver com várias famílias ou membros da família.)

Page 2<br>Item 9	Has your child ever experienced verbal or physical abuse or threats from a romantic partners? (for example, a boyfriend or girlfriend)	Seu filho(a) já sofreu abuso verbal ou físico ou ameaças de um parceiro romântico de um de seus pais/responsável?<br>*(por exemplo, um namorado ou namorada* de um de seus pais / responsável).	J3: The original question conveys the idea that the boyfriend/girlfriend referred to is the adolescent's partner, not that of the parents or guardians.	The text has been modified with the agreement of the translators and judges to align with the original text.	Seu filho(a) já sofreu abuso verbal ou físico ou ameaças de um parceiro romântico?<br>(Por exemplo, um namorado ou namorada.)
