# Supplementary material for: Pediatrics ACES and related life event screener (PEARLS): translation, transcultural adaptation, and validation to Brazilian Portuguese
Source: J Pediatr (Rio J). 2024 Oct 29;101(2):262–8. doi: 10.1016/j.jped.2024.10.003 (PMC11889689; doi:10.1016/j.jped.2024.10.003)
Supplement: Supplementary file 3 [file mmc3.pdf]

GUIDELINES:

Please read each item aloud and provide a brief oral explanation of the meaning of each item. If the language does not seem appropriate, you may suggest modifications, alternatives, or synonyms, but please note the reason for your suggestion.

Use the table below for your evaluation:

<table>
<tr><th colspan="2" rowspan="2">Items</th><th colspan="2">Mark with an X if you agree</th><th rowspan="2">Synonyms or suggestion for change</th><th rowspan="2">Reason for change</th></tr>
<tr><th>Clear, Understandable, and Well-Written</th><th>Appropriate terms that align with reality</th></tr>
<tr><td colspan="2">Cover Instructions</td><td></td><td></td><td></td><td></td></tr>
<tr><td colspan="2">Page 1 Instructions in the Header</td><td></td><td></td><td></td><td></td></tr>
<tr><td rowspan="10">Page 1 – part 1 – question</td><td>1</td><td></td><td></td><td></td><td></td></tr>
<tr><td>2</td><td></td><td></td><td></td><td></td></tr>
<tr><td>3</td><td></td><td></td><td></td><td></td></tr>
<tr><td>4</td><td></td><td></td><td></td><td></td></tr>
<tr><td>5</td><td></td><td></td><td></td><td></td></tr>
<tr><td>6</td><td></td><td></td><td></td><td></td></tr>
<tr><td>7</td><td></td><td></td><td></td><td></td></tr>
<tr><td>8</td><td></td><td></td><td></td><td></td></tr>
<tr><td>9</td><td></td><td></td><td></td><td></td></tr>
<tr><td>10</td><td></td><td></td><td></td><td></td></tr>
<tr><td colspan="2">Page 1 footer</td><td></td><td></td><td></td><td></td></tr>
<tr><td rowspan="9">Page 2 - part 2 – question</td><td>1</td><td></td><td></td><td></td><td></td></tr>
<tr><td>2</td><td></td><td></td><td></td><td></td></tr>
<tr><td>3</td><td></td><td></td><td></td><td></td></tr>
<tr><td>4</td><td></td><td></td><td></td><td></td></tr>
<tr><td>5</td><td></td><td></td><td></td><td></td></tr>
<tr><td>6</td><td></td><td></td><td></td><td></td></tr>
<tr><td>7</td><td></td><td></td><td></td><td></td></tr>
<tr><td>8</td><td></td><td></td><td></td><td></td></tr>
<tr><td>9</td><td></td><td></td><td></td><td></td></tr>
</table>

Profession of the Responding Professional: ______________________________

<table>
<tr><td colspan="2">Page 2 footer</td><td></td><td></td><td></td><td></td></tr>
<tr><td rowspan="3">Page 3 - part 3 – question</td><td>Instructions</td><td></td><td></td><td></td><td></td></tr>
<tr><td>1</td><td></td><td></td><td></td><td></td></tr>
<tr><td>2</td><td></td><td></td><td></td><td></td></tr>
</table>

Profession of the Responding Professional: ____
