# Supplementary material for: Pediatrics ACES and related life event screener (PEARLS): translation, transcultural adaptation, and validation to Brazilian Portuguese
Source: J Pediatr (Rio J). 2024 Oct 29;101(2):262–8. doi: 10.1016/j.jped.2024.10.003 (PMC11889689; doi:10.1016/j.jped.2024.10.003)
Supplement: Supplementary file 4 [file mmc4.pdf]

Table with Suggested Changes Based on Feedback (Face validity) from the Target Audience

Target Audience	Suggestion	Reason	Evaluation of the Suggestion
**Health Professional**	Page 1 - Question 3<br>add "*xingou*".	To adopt a more colloquial language.	In the original, the word "insulted" is used, which the professors who performed the translation translated as "*insultou*." It is understood that "*insultou*" is easily comprehensible for the population whose official language is Portuguese.
**Health Professional**	Page 1 – Header Instructions<br>Include an example in the header.	I felt the need to look at the question to better understand the instruction.	It is understood that there is no need to include the example in the header because right below there are already questions that serve as examples for understanding what is being explained in the header.
**Underdegree student**	Page 1 - Question 3<br>Change "*insultar*" to "*xingou*".	The person did not say.	In the original, the word "insulted" is used, which the professors who performed the translation rendered as "*insultou*". It is understood that "*insultou*" is easily comprehensible for the population whose official language is Portuguese.
**Underdegree student**	Page 1 – Header Instructions - Put "the entire question" in bold.<br><br>Page 2 – Question 3<br>" *Execução Hipotecária*".	To highlight the information.<br><br>The term could be replaced with something easier.	In the original, there is no emphasis in the header. To avoid visually cluttering the document, the suggestion for bold text will not be implemented.<br><br>In the original, the word "foreclosure" is used, which translates to "*execução hipotecária*" in Portuguese. When searching for a synonym in the Portuguese dictionary, no term was found that could be easier to substitute.
**Underdegree student**	**Cover**<br>*experiência → vivência*.	The term "*experienciam*" in the previous line is repetitive.	Since the original uses the word "experience" in the preceding sentence and "experiences" in the following sentence, we will keep the terms "*experienciam*" and "*experiências*" in the Portuguese version. It is understood that this remains clear and comprehensible for the reader.

**Underdegree student**	Page 1 – question 10<br>status → *relacionamento amoroso.*	Understanding of the Adolescent	It is understood that "relationship status" is different from "romantic relationship," where the former refers to legal/juridical matters and practical aspects, while the latter involves feelings between two people.
	Page 2 – Question 5<br>Sheltering situations in shelters or reformatories	Understanding of the Adolescent	"*Acolhimento familiar*" is a broader concept. "*Abrigo*" and "*reformatório*"are types of institutional care.
