# Supplementary material for: Pediatrics ACES and related life event screener (PEARLS): translation, transcultural adaptation, and validation to Brazilian Portuguese
Source: J Pediatr (Rio J). 2024 Oct 29;101(2):262–8. doi: 10.1016/j.jped.2024.10.003 (PMC11889689; doi:10.1016/j.jped.2024.10.003)
Supplement: Supplementary file 5 [file mmc5.pdf]

Supplementary Material 5: Adaptations of the Pediatric ACEs and Relevant Life Events Screener (PEARLS) in the translation and transcultural adaptation to Brazilian Portuguese process[a].

Item	Original	S1	S2	S1TR	Final Version
**Title on the cover**	PEARLS<br><br>Pediatrics ACEs and Related Life Events Screener<br><br>Teen	PEARLS<br><br>Experiências Adversas na Infância Pediátrico e Rastreador de Eventos de Vida Relacionados<br><br>ADOLESCENTE	PEARLS<br><br>Experiências Adversas na Infância Pediátrico e Rastreador de Eventos de Vida Relacionados<br><br>ADOLESCENTE	PEARLS<br><br>Pediatrics Adverse Childhood Experiences and Related Life Events Screener<br><br>Teen	PEARLS-BR<br><br>Experiências Adversas na Infância Pediátrico e Rastreador de Eventos de Vida Relacionados<br><br>ADOLESCENTE
**Instruction on the cover**	Many families experience stressful events in their lives. Over time these experiences may affect your child's health and wellbeing. There are many things you can do and may already be doing to help. Because these events are so common, we are offering resources to all caregivers in our clinic on how to support your child and yourself. We are asking everyone these questions. Some people find that answering the questions gives you time to think about how certain experiences may be impacting your child's health and what you can do to help. Your answers help us to support you and your child to be as healthy as possible. The survey is confidential and optional*. Your experiences are your own and no matter what you choose to share, we are here to help.	Muitas famílias experienciam eventos estressantes ao longo de suas vidas. Com o tempo, essas experiências podem afetar a saúde e o bem estar do seu filho(a). Existem muitas coisas que você pode fazer ou talvez já esteja fazendo para ajudar. Como esses eventos são muito comuns, estamos oferecendo recursos a todos os cuidadores em nossa clínica sobre como apoiar seu filho(a) e você mesmo. Estamos fazendo essas perguntas para todos(as). Para algumas pessoas, responder as questões ajuda a pensar sobre como certas experiências podem impactar a saúde do seu filho(a) e o que você pode fazer para ajudar. Suas respostas nos ajudam a apoiar você e seu filho a serem o mais saudáveis possível. A pesquisa é confidencial e opcional*. As experiências são suas e não importa o que você	Muitas famílias experienciam eventos estressantes ao longo de suas vidas. Com o tempo, essas experiências podem afetar a saúde e o bem estar do seu filho(a). Existem muitas coisas que você pode fazer ou talvez já esteja fazendo para ajudar. Como esses eventos são muito comuns, estamos oferecendo recursos a todos os cuidadores em nossa clínica sobre como apoiar seu filho(a) e você mesmo.<br>Estamos fazendo essas perguntas para todos(as). Para algumas pessoas, responder as questões ajuda a pensar sobre como certas experiências podem impactar a saúde do seu filho(a) e o que você pode fazer para ajudar. Suas respostas nos ajudam a apoiar você e seu filho a serem o mais saudáveis possível. A pesquisa é confidencial e opcional*.<br>As experiências são suas e não importa o que você escolha	Many families experience stressful events throught their lives. Over time, these experiences can affect your health and well-being.<br>There are many things that you can do or that may you are already doing to help. As these events are very common, we are offering resources to all the caregivers in our clinic about how to support you.<br>We are asking everyone these questions. For some people, answering the questions helps them to think about how certain experiences might impact your health.<br>Your answers help us to support you to be as healthy as possible. The survey is confidential and optional*. The experiences are yours and no matter what you choose to share, we're here to help.<br>*It is important that you know that, if at this time you are not safe, or is being physically,	Muitas famílias experienciam eventos estressantes ao longo de suas vidas. Com o tempo, essas experiências podem afetar a saúde e o bem estar do seu filho(a). Existem muitas coisas que você pode fazer ou talvez já esteja fazendo para ajudar. Como esses eventos são muito comuns, estamos oferecendo recursos a todos os cuidadores em nossa clínica sobre como apoiar seu filho(a) e você mesmo.<br>Estamos fazendo essas perguntas para todos(as). Para algumas pessoas, responder as questões ajuda a pensar sobre como certas experiências podem impactar a saúde do seu filho(a) e o que você pode fazer para ajudar. Suas respostas nos ajudam a apoiar você e seu filho a serem o mais saudáveis possível. A pesquisa é confidencial e opcional*.<br>As experiências são suas e não importa o que você escolha

	*It is important for you to know though that if you tell us that your child is currently unsafe or being physically, verbally, or sexually hurt or neglected we may need to share this with other authorities.	escolha compartilhar, nós estamos aqui para ajudar.<br>*Porém, é importante saber que caso você nos conte que no momento seu filho não está seguro, ou está sendo abusado ou negligenciado física, verbal ou sexualmente, nós possivelmente teremos que compartilhar essas informações com as autoridades.	compartilhar, nós estamos aqui para ajudar.<br>*Porém, é importante saber que caso você nos conte que no momento seu filho não está seguro, ou está sendo abusado ou negligenciado física, verbal ou sexualmente, nós possivelmente teremos que compartilhar essas informações com as autoridades.	verbally, or sexually abused or neglected, we will possibly have to share this information with the authorities.	compartilhar, nós estamos aqui para ajudar.<br>*Porém, é importante saber que caso você nos conte que no momento seu filho não está seguro, ou está sendo abusado ou negligenciado física, verbal ou sexualmente, nós possivelmente teremos que compartilhar essas informações com as autoridades.
**Page 1 Title**	Pediatric ACEs and Related Life Events Screener (PEARLS)	Experiências Adversas Na Infância Pediátrico e Rastreador De Eventos De Vida Relacionados (PEARLS)	Experiências Adversas na Infância Pediátrico e Rastreador De Eventos De Vida Relacionados (PEARLS)	Pediatric Adverse Childhood Experiences and Related Life Events Screener (PEARLS-BR)	Experiências Adversas na Infância Pediátrico e Rastreador De Eventos De Vida Relacionados (PEARLS-BR)
**Page 1 Sentence under the title**	TEEN - To be completed by: Caregiver	ADOLESCENTE – deve ser preenchido pelo: PAI/MÃE/RESPONSÁVEL	ADOLESCENTE – deve ser preenchido pelo: PAI/MÃE/RESPONSÁVEL	ADOLESCENT – must be filled out by: father/mother/guardian	ADOLESCENTE – deve ser preenchido pelo: PAI/MÃE/RESPONSÁVEL
**Page 1 Instruction**	At any point in time since your child was born, has your child seen or been present when the following experiences happened? Please include past and present experiences.<br>Please note, some questions have more than one part separated by “OR.” If any part of the question is answered “Yes,” then the answer to the entire question is “Yes.”	Em algum momento desde o nascimento de seu filho(a), ele/ela viu ou esteve presente quando as seguintes experiências aconteceram? Inclua experiências passadas e presentes.<br>Observe que algumas perguntas têm mais de uma parte separada por “OU”. Se alguma parte da pergunta for respondida "Sim", a resposta a toda a pergunta é "Sim".	Em algum momento desde o nascimento de seu filho(a), ele/ela viu ou esteve presente quando as seguintes experiências aconteceram? Inclua experiências passadas e presentes.<br>Observe que algumas perguntas têm mais de uma parte separada por “OU”. Se alguma parte da pergunta for respondida "Sim", a resposta a toda a pergunta é "Sim".	At any point since the birth of your child, has your son or daughter been present when the following experiences happened? Include past and present experiences.<br>Note that some questions have more than one part separated by “OR”. If the answer to any part of the question is "Yes”, the answer to the whole question is "Yes".	Em algum momento desde o nascimento de seu filho(a), ele/ela viu ou esteve presente quando as seguintes experiências aconteceram? Inclua experiências passadas e presentes.<br>Por favor, observe que algumas perguntas têm mais de uma parte separada por “OU”. Se alguma parte da pergunta for respondida "Sim", a resposta a toda a pergunta é "Sim".
**Page 1 Part 1 Item 1**	Do you think your child ever felt unsupported, unloved and/or unprotected?	Você acha que seu filho(a) já se sentiu sem apoio, sem amor e / ou desprotegido?	Você acha que seu filho(a) já se sentiu sem apoio, sem amor e / ou desprotegido?	Do you think that your child has ever felt a lack of support, unloved, and/or unprotected?	Você acha que seu filho(a) já se sentiu sem apoio, sem amor e / ou desprotegido?

**Page 1<br>Part 1<br>Item 2**	Has your child ever lived with a parent/caregiver who had mental health issues?<br>(For example, depression, schizophrenia, bipolar disorder, PTSD, or an anxiety disorder)	Seu filho(a) já morou com um dos pais/ responsável que apresentava problemas de saúde mental?	Seu filho(a) já morou com um dos pais/responsável que apresentava problemas de saúde mental?<br>(Por exemplo, depressão, esquizofrenia, transtorno bipolar, transtorno de estresse pós-traumático e/ou transtorno de ansiedade.)	Has your child ever lived with a parent/guardians that experienced mental health problems?<br>(for example, depression, schizophrenia, bipolar disorder, post-traumatic stress disorder and/or anxiety disorder).	Seu filho(a) já morou com um dos pais/responsável que apresentava problemas de saúde mental?<br>(Por exemplo, depressão, esquizofrenia, transtorno bipolar, transtorno de estresse pós-traumático e/ou transtorno de ansiedade.)
**Page 1<br>Part 1<br>Item 3**	Has a parent/caregiver ever insulted, humiliated, or put down your child?	Algum dos pais/responsável já insultou, humilhou ou rebaixou seu filho(a)?	Algum dos pais/responsável já insultou, humilhou ou rebaixou seu filho(a)?	Has any parent/guardian ever insulted, humiliated or demeaned your child?	Algum dos pais/responsável já insultou, humilhou ou rebaixou seu filho(a)?
**Page 1<br>Part 1<br>Item 4**	Has the child’s biological parent or any caregiver ever had, or currently has a problem with too much alcohol, street drugs or prescription medications use?	Um dos pais biológicos da criança ou algum responsável já teve, ou atualmente tem um problema com o uso excessivo de álcool, drogas ou medicamentos prescritos?	Um dos pais biológicos da criança ou algum responsável já teve, ou atualmente tem um problema com o uso excessivo de álcool, drogas ou medicamentos prescritos?	Has a biological parent or guardian ever had, or currently has any problem with excessive use of alcohol, drugs, or prescription medications?	Um dos pais biológicos da criança ou algum responsável já teve, ou atualmente tem um problema com o uso excessivo de álcool, drogas ou medicamentos prescritos?
**Page 1<br>Part 1<br>Item 5**	Has your child ever lacked appropriate care by any caregiver?<br>(For example, not being protected from unsafe situations, or not cared for when sick or injured even when the resources were available)	Seu filho(a) já teve falta de cuidados adequados por parte de algum responsável?<br>(por exemplo, não ser protegido de situações inseguras, ou não ser cuidado quando doente ou ferido, mesmo quando os recursos estavam disponíveis).	Seu filho(a) já teve falta de cuidados adequados por parte de algum responsável?<br>(por exemplo, não ser protegido de situações inseguras, ou não ser cuidado quando doente ou ferido, mesmo quando os recursos estavam disponíveis).	Has your child ever experienced a lack of adequate care on the part of a parent/guardian?<br>(for example, not being protected in unsafe situations, or not being taken care of when sick or injured, even when the resources were available).	Seu filho(a) já teve falta de cuidados adequados por parte de algum responsável?<br>(por exemplo, não ser protegido de situações inseguras, ou não ser cuidado quando doente ou ferido, mesmo quando os recursos estavam disponíveis).
**Page 1<br>Part 1<br>Item 6**	Has your child seen or heard a parent/caregiver:<br>(Mark yes, if any are true for you or your family)<br>screamed at, sworn at, insulted, or humiliated by someone they know?<br>ever slapped, kicked, punched, beaten up or hurt with a weapon?	Seu filho já viu ou ouviu um dos pais/responsável: (assinale sim, se qualquer um for verdadeiro para você ou sua família).<br><br>sendo gritado(a), xingado(a), insultado(a) ou humilhado(a) por outro adulto?<br>sendo esbofeteado(a), chutado(a), socado(a), espancado(a) ou ferido(a) com uma arma?	Seu filho(a) já viu ou ouviu um dos pais/responsável:<br>(Assinale sim, se qualquer um for verdadeiro para você ou sua família).<br>Sendo tratado a gritos ou xingado(a), insultado(a) ou humilhado(a) por outro adulto?<br>OU sendo esbofeteado(a), chutado(a), socado(a), espancado(a) ou ferido(a) com uma arma?	Has your child ever seen/heard a parent/guardian:<br>(Mark “yes”, if any are true for you or your family).<br>Being screamed or sworn at, insulted, or humiliated by another adult?<br>OR being slapped, kicked, punched, beaten, or injured with a weapon?	Seu filho(a) já viu ou ouviu um dos pais/responsável:<br>(Assinale sim, se qualquer um for verdadeiro para você ou sua família).<br>Sendo tratado a gritos ou xingado(a), insultado(a) ou humilhado(a) por outro adulto?<br>OU sendo esbofeteado(a), chutado(a), socado(a), espancado(a) ou ferido(a) com uma arma?

**Page 1 Part 1 Item 7**	Has any adult in the household: (Mark yes, if any are true for you or your family) often or very often pushed, grabbed, slapped, or thrown something at your child? ever hit your child so hard that your child had marks or was injured? ever threatened your child or acted in a way that made your child afraid that they might be hurt?	Algum adulto na casa (assinale sim, se qualquer um for verdadeiro para você ou sua família). frequentemente ou muito frequentemente empurrou, agarrou, deu um tapa ou jogou algo em seu filho(a)? já bateu em seu filho(a) com tanta força que deixou marcas ou o/a machucou? já ameaçou seu filho(a) ou agiu de maneira que o/a fez ficar com medo de se machucar?	Algum adulto na casa (assinale sim, se qualquer um for verdadeiro para você ou sua família). frequentemente ou muito frequentemente empurrou, agarrou, deu um tapa ou jogou algo em seu filho(a)? OU já bateu em seu filho(a) com tanta força que deixou marcas ou o/a machucou? OU já ameaçou seu filho(a) ou agiu de maneira que o/a fez ficar com medo de se machucar?	Has any adult at household: (Mark “yes” if any are true for you or your family). Frequently or very frequently pushed, grabbed, spanked, or thrown something at your child? OR ever hit your child with such force that it left marks or hurt your child? OR ever threatened your child or acted in such a way that it made your child afraid of being hurt?	Algum adulto na casa (assinale sim, se qualquer um for verdadeiro para você ou sua família). frequentemente ou muito frequentemente empurrou, agarrou, deu um tapa ou jogou algo em seu filho(a)? OU já bateu em seu filho(a) com tanta força que deixou marcas ou o/a machucou? OU já ameaçou seu filho(a) ou agiu de maneira que o/a fez ficar com medo de se machucar?
**Page 1 Part 1 Item 8**	Has your child ever experienced sexual abuse? (For example, anyone touched your child or asked your child to touch that person in a way that was unwanted, or made your child feel uncomfortable, or anyone ever attempted or actually had oral, anal, or vaginal sex with your child)	Seu filho(a) já sofreu abuso sexual? (por exemplo, alguém tocou em seu filho(a) ou pediu a ele/ela que tocasse essa pessoa de uma forma indesejada ou fez seu filho(a) se sentir desconfortável, ou alguém já tentou ou realmente fez sexo oral, anal ou vaginal com seu/sua filho(a))	Seu filho(a) já sofreu abuso sexual? (por exemplo, alguém tocou em seu filho(a) ou pediu a ele/ela que tocasse essa pessoa de uma forma indesejada ou fez seu filho(a) se sentir desconfortável, ou alguém já tentou ou realmente fez sexo oral, anal ou vaginal com seu/sua filho(a))	Has your child ever experienced sexual abuse? (Por example, anybody touched your child or asked him/her to touch that person in an unwanted way or made your child feel uncomfortable, or someone attempted or actually performed oral, anal or vaginal sex with your child.)	Seu filho(a) já sofreu abuso sexual? (por exemplo, alguém tocou em seu filho(a) ou pediu a ele/ela que tocasse essa pessoa de uma forma indesejada ou fez seu filho(a) se sentir desconfortável, ou alguém já tentou ou realmente fez sexo oral, anal ou vaginal com seu/sua filho(a))
**Page 1 Part 1 Item 9**	Has your child ever lived with a parent/caregiver who went to jail/prison?	Seu filho(a) já morou com um dos pais/responsável que foi para a cadeia/ prisão?	Seu filho(a) já morou com um dos pais/responsável que foi para a cadeia/ prisão?	Has your child ever lived with parents/guardians that went to jail/prison?	Seu filho(a) já morou com um dos pais/responsável que foi para a cadeia/ prisão?
**Page 1 Part 1 Item 10**	Have there ever been big changes in the relationship status of the child’s caregiver(s)? (for example, a parent/caregiver got a divorce or separated, or a romantic partner moved in or out)	Já houve mudanças significativas no status de relacionamento do(s) responsável(eis) da criança? (por exemplo, um dos pais/responsável se divorciou ou se separou, ou um parceiro romântico se mudou para a mesma residência ou saiu da mesma?)	Já houve mudanças significativas no status de relacionamento do(s) responsável(eis) da criança? (por exemplo, um dos pais/responsável se divorciou ou se separou, ou um parceiro romântico se mudou para a mesma residência ou saiu da mesma?)	Have there been significant changes in the relationship status of the child’s parents/guardians? (for example, parents/guardians got divorced or separated, or a romantic partner moved into or out of the same residence?)	Já houve mudanças significativas no status de relacionamento do(s) responsável(eis) da criança? (por exemplo, um dos pais/responsável se divorciou ou se separou, ou um parceiro romântico se mudou para a mesma residência ou saiu da mesma?)

**Page 1 Footer**	How many "Yes" did you answer in Part 1?<br><br>Please continue to the other side for the rest of questionnaire.	Quantos "Sim" você respondeu na Parte 1?<br><br>Por favor continue respondendo o restante do questionário do outro lado da página.	Quantos "Sim" você respondeu na Parte 1?<br><br>Por favor continue respondendo o restante do questionário do outro lado da página.	How many times have you marked "Yes" in Part 1?<br><br>Please continue answering the resto f the questionnaire on the other side of the page.	Quantos "Sim" você respondeu na Parte 1?<br><br>Por favor continue respondendo o restante do questionário do outro lado da página.
**Page 2 Part 2 Item 1**	Has your child ever seen, heard, or been a victim of violence in your neighborhood, community, or school? (For example, targeted bullying, assault, or other violent actions, war or terrorism).	Seu filho(a) já viu, ouviu ou foi vítima de violência em seu bairro, comunidade ou escola? (por exemplo, bullying direcionado, agressão ou outras ações violentas, guerra ou terrorismo).	Seu filho(a) já viu, ouviu ou foi vítima de violência em seu bairro, comunidade ou escola? (Por exemplo, bullying direcionado, agressão ou outras ações violentas, guerra ou terrorismo).	Has your child ever seen, heard or been a victim of violence in your neighborhood, community, or school? (For example, targeted bullying, aggression or other violent acts, war or terrorism).	Seu filho(a) já viu, ouviu ou foi vítima de violência em seu bairro, comunidade ou escola? (Por exemplo, bullying direcionado, agressão ou outras ações violentas, guerra ou terrorismo).
**Page 2 Part 2 Item 2**	Has your child experienced discrimination? (for example, being hassled or made to feel inferior or excluded because of their race, ethnicity, gender identity, sexual orientation, religion, learning differences, or disabilities).	Seu filho(a) já experienciou discriminação? (por exemplo, foi incomodado ou se sentiu inferior ou excluído devido à sua raça, etnia, identidade de gênero, orientação sexual, religião, dificuldades de aprendizagem ou deficiências).	Seu filho(a) já experienciou discriminação? (Por exemplo, foi incomodado ou se sentiu inferior ou excluído devido à sua raça, etnia, identidade de gênero, orientação sexual, religião, dificuldades de aprendizagem ou deficiências.)	Has your child ever experienced discrimination? (for example, being disturbed or made to feel inferior or excluded due to your child's race, ethnicity, gender identity, sexual orientation, religion, learning difficulties or disabilities.)	Seu filho(a) já experienciou discriminação? (Por exemplo, foi incomodado ou se sentiu inferior ou excluído devido à sua raça, etnia, identidade de gênero, orientação sexual, religião, dificuldades de aprendizagem ou deficiências.)
**Page 2 Part 2 Item 3**	Has your child ever had problems with housing? (For example, being homeless, not having a stable place to live, moved more than two times in a six-month period, faced eviction or foreclosure, or had to live with multiple families or family members).	Seu filho(a) já teve problemas de moradia? (por exemplo, ser sem-teto, não ter um lugar estável para morar, se mudar mais de duas vezes em um período de seis meses, enfrentou despejo ou execução hipotecária ou teve que viver com várias famílias ou membros da família)	Seu filho(a) já teve problemas de moradia? (Por exemplo, ser sem-teto, não ter um lugar estável para morar, se mudar mais de duas vezes em um período de seis meses, enfrentou despejo ou execução hipotecária ou teve que viver com várias famílias ou membros da família.)	Has your child ever had problems with housing? (for example, being homeless, not having a stable place to live, having to move more than twice during a period of six months, facing eviction or foreclosure or having to live with various families or family members).	Seu filho(a) já teve problemas de moradia? (Por exemplo, ser sem-teto, não ter um lugar estável para morar, se mudar mais de duas vezes em um período de seis meses, enfrentou despejo ou execução hipotecária ou teve que viver com várias famílias ou membros da família.)

**Page 2 Part 2 Item 4**	Are you currently or have you ever:<br>(Mark yes, if any apply):<br>worried that the food for your child would run out before you got money to buy more?<br>had the food you bought for your child not last and did not have money to buy more?	Você atualmente ou alguma vez: (assinale sim, se qualquer um for verdadeiro para você ou sua família):<br><br>Ficou preocupado que a comida para seu filho(a) acabasse antes de você tivesse dinheiro para comprar mais?<br>Que a comida que você comprou para seu filho(a) não durou e não teve dinheiro para comprar mais?	Você atualmente ou alguma vez: (assinale sim, se qualquer um for verdadeiro para você ou sua família):<br><br>Ficou preocupado que a comida para seu filho(a) acabasse antes de você tivesse dinheiro para comprar mais?<br>OU que a comida que você comprou para seu filho(a) não durou e não teve dinheiro para comprar mais?	Are you currently or have you ever:<br>(Mark yes, if any apply):<br>Been worried that the food for your child would run out before you could buy more?<br>OR had the food you bought for your child not last and did not have money to buy more?	Você atualmente ou alguma vez: (assinale sim, se qualquer um for verdadeiro para você ou sua família):<br><br>Ficou preocupado que a comida para seu filho(a) acabasse antes de você tivesse dinheiro para comprar mais?<br>OU que a comida que você comprou para seu filho(a) não durou e não teve dinheiro para comprar mais?
**Page 2 Part 2 Item 5**	Has your child ever been separated from their parent or caregiver due to foster care, or immigration?	Seu filho(a) já foi separado(a) de seus pais ou responsáveis devido a situações de acolhimento familiar ou imigração?	Seu filho(a) já foi separado(a) de seus pais ou responsáveis devido a situações de acolhimento familiar ou imigração?	Has your child ever been separated from their parents or guardians due to foster care or immigration situations?	Seu filho(a) já foi separado(a) de seus pais ou responsáveis devido a situações de acolhimento familiar ou imigração?
**Page 2 Part 2 Item 6**	Has your child ever lived with a parent/caregiver who had a serious physical illness or disability?	O seu filho(a) já morou com um dos pais / responsável que sofre de uma doença física grave ou deficiência?	O seu filho(a) já morou com um dos pais / responsável que sofre de uma doença física grave ou deficiência?	Has your child ever lived with any of their parents/guardians that suffers from a serious physical illness or disability?	O seu filho(a) já morou com um dos pais / responsável que sofre de uma doença física grave ou deficiência?
**Page 2 Part 2 Item 7**	Has your child ever lived with a parent or caregiver who died?	Seu filho(a) já morou com um dos pais ou responsável que tenha falecido?	Seu filho(a) já morou com um dos pais ou responsável que tenha falecido?	Has your child ever lived with a parent or guardian who died?	Seu filho(a) já morou com um dos pais ou responsável que tenha falecido?
**Page 2 Part 2 Item 8**	Has your child ever been detained, arrested or incarcerated?	Seu filho(a) já foi detido(a), preso(a) ou encarcerado(a)?	Seu filho(a) já foi detido(a), preso(a) ou encarcerado(a)?	Has your child ever been detained, arrested, or incarcerated?	Seu filho(a) já foi detido(a), preso(a) ou encarcerado(a)?
**Page 2 Part 2 Item 9**	Has your child ever experienced verbal or physical abuse or threats from a romantic partners? (for example, a boyfriend or girlfriend)	Seu filho(a) já sofreu abuso verbal ou físico ou ameaças de um parceiro romântico de um de seus pais/responsável?<br>(por exemplo, um namorado ou namorada de um de seus pais / responsável).	Seu filho(a) já sofreu abuso verbal ou físico ou ameaças de um parceiro romântico?<br>(Por exemplo, um namorado ou namorada.)	Has your child ever suffered verbal or physical abuse or threats from a romantic partner?<br>(For example, a boyfriend or girlfriend).	Seu filho(a) já sofreu abuso verbal ou físico ou ameaças de um parceiro romântico?<br>(Por exemplo, um namorado ou namorada.)
**Page 3 Instruction**	We would like to understand more about your child's and your family's strengths and resources.	PARTE 3: Gostaríamos de entender mais sobre os pontos fortes do seu filho(a) e da sua família.	PARTE 3: Gostaríamos de entender mais sobre os pontos fortes do seu filho(a) e da sua família.	Part 3:<br>We would like to understand more about the resources and strengths of your child and of your family.	PARTE 3: Gostaríamos de entender mais sobre os pontos fortes do seu filho(a) e da sua família.

**Page 3 Item 1**	What are your child's best qualities?	Quais são as melhores qualidades do seu filho(a)?	Quais são as melhores qualidades do seu filho(a)?	What are your child's best qualities?	Quais são as melhores qualidades do seu filho(a)?
**Page 3 item 2**	What are things that help you (or your family) get through hard times (or cope with stress)?	Quais são as coisas que ajudam você (ou sua família) a superar os momentos difíceis (ou lidar com o estresse)?	Quais são as coisas que ajudam você (ou sua família) a superar os momentos difíceis (ou lidar com o estresse)?	What are the things that help you (or your family) to overcome difficult moments (or deal with stress)?	Quais são as coisas que ajudam você (ou sua família) a superar os momentos difíceis (ou lidar com o estresse)?

[a] The PEARLS TEEN and PEARLS TEEN SELF-REPORT instruments comprise nine questions in part 2, while the PEARLS CHILD version concludes at question 7 in the same sectio
