# Supplementary material for: Pediatrics ACES and related life event screener (PEARLS): translation, transcultural adaptation, and validation to Brazilian Portuguese
Source: J Pediatr (Rio J). 2024 Oct 29;101(2):262–8. doi: 10.1016/j.jped.2024.10.003 (PMC11889689; doi:10.1016/j.jped.2024.10.003)
Supplement: Supplementary file 6 [file mmc6.pdf]

PEARLS-BR

Experiências Adversas na Infância Pediátrico e Rastreador de Eventos de Vida Relacionados

Criança

Muitas famílias experienciam eventos estressantes ao longo de suas vidas. Com o tempo, essas experiências podem afetar a saúde e o bem estar do seu filho(a). Existem muitas coisas que você pode fazer ou talvez já esteja fazendo para ajudar. Como esses eventos são muito comuns, estamos oferecendo recursos a todos os cuidadores em nossa clínica sobre como apoiar seu filho(a) e você mesmo.

Estamos fazendo essas perguntas para todos(as). Para algumas pessoas, responder as questões ajuda a pensar sobre como certas experiências podem impactar a saúde do seu filho(a) e o que você pode fazer para ajudar. Suas respostas nos ajudam a apoiar você e seu filho a serem o mais saudáveis possível. A pesquisa é confidencial e opcional*.

As experiências são suas e não importa o que você escolha compartilhar, nós estamos aqui para ajudar.

** Porém, é importante saber que caso você nos conte que, no momento, seu filho(a) não está seguro, ou está sendo abusado ou negligenciado física, verbal ou sexualmente, nós possivelmente teremos que compartilhar essas informações com as autoridades.*

Aviso de direitos autorais e isenção de responsabilidade: Certos materiais aqui incorporados são Copyright © 2016 e 2018, *The Regents of the University of California*. Todos os direitos reservados.

2024 Universidade de Caxias do Sul. Tradução, adaptação brasileira e validação.

Experiências Adversas na Infância Pediátrico e Rastreador de Eventos de Vida Relacionados (PEARLS - BR)

CRIANÇA – deve ser preenchido pelo: **PAI/MÃE/RESPONSÁVEL**

Em algum momento desde o Nascimento de seu filho(a), ele/ela viu ou esteve presente quando as seguintes experiências aconteceram? Inclua experiências passadas e presentes.

Por favor, observe que algumas perguntas têm mais de uma parte separada por "OU". Se alguma parte da pergunta for respondida "Sim", a resposta a toda a pergunta é "Sim".

**PARTE 1:**	**Marque "SIM" onde se aplica**	X
**1.**	Você acha que seu filho(a) já se sentiu sem apoio, sem amor e/ou desprotegido?	☐
**2.**	Seu filho(a) já morou com um dos pais/responsável que apresentava problemas de saúde mental?<br>*(Por exemplo, depressão, esquizofrenia, transtorno bipolar, transtorno de estresse pós-traumático e/ou transtorno de ansiedade.)*	☐
**3.**	Algum dos pais/responsável já insultou, humilhou ou rebaixou seu filho(a)?	☐
**4.**	Um dos pais biológicos da criança ou algum responsável já teve, ou atualmente tem um problema com o uso excessivo de álcool, drogas ou medicamentos prescritos?	☐
**5.**	Seu filho(a) já teve falta de cuidados adequados por parte de algum responsável?<br>*(Por exemplo, não ser protegido de situações inseguras, ou não ser cuidado quando doente ou ferido, mesmo quando os recursos estavam disponíveis.)*	☐
**6.**	Seu filho(a) já viu ou ouviu um dos pais/responsável:<br>*(Assinale sim, se qualquer um for verdadeiro para você ou sua família).*<br>**a.** Sendo tratado a gritos ou xingado(a), insultado(a) ou humilhado(a) por outro adulto?<br>**b.** OU sendo esbofeteado(a), chutado(a), socado(a), espancado(a) ou ferido(a) com uma arma?	☐
**7.**	Algum adulto na casa:<br>(Assinale sim, se qualquer um for verdadeiro para você ou sua família).<br>**a.** Frequentemente ou muito frequentemente empurrou, agarrou, deu um tapa ou jogou algo em seu filho(a)?<br>**b.** OU já bateu em seu filho(a) com tanta força que deixou marcas ou o/a machucou?<br>**c.** OU já ameaçou seu filho(a) ou agiu de maneira que o/a fez ficar com medo de se machucar?	☐
**8.**	Seu filho(a) já sofreu abuso sexual?<br>*(Por exemplo, alguém tocou em seu filho(a) ou pediu a ele/ ela que tocasse essa pessoa de uma forma indesejada ou fez seu filho(a) se sentir desconfortável, ou alguém já tentou ou realmente fez sexo oral, anal ou vaginal com seu filho(a).)*	☐
**9.**	Seu filho(a) já morou com um dos pais/responsável que foi para a cadeia/prisão?	☐
**10.**	Já houve mudanças significativas no status de relacionamento do(s) responsável(eis) da criança?<br>*(Por exemplo, um dos pais/responsável se divorciou ou se separou, ou um parceiro romântico se mudou para a mesma residência ou saiu da mesma.)*	☐

Quantos "Sim" você respondeu na Parte 1? ☐

Por favor continue respondendo o restante do questionário do outro lado da página. →

PARTE 2:	Marque “SIM” onde se aplica	X
**1.**	Seu filho(a) já viu, ouviu ou foi vítima de violência em seu bairro, comunidade ou escola?<br>(Por exemplo, bullying direcionado, agressão ou outras ações violentas, guerra ou terrorismo).	☐
**2.**	Seu filho(a) já experienciou discriminação?<br>*(Por exemplo, foi incomodado ou se sentiu inferior ou excluído devido à sua raça, etnia, identidade de gênero, orientação sexual, religião, dificuldades de aprendizagem ou deficiências.)*	☐
**3.**	Seu filho(a) já teve problemas de moradia?<br>(*Por exemplo, ser sem-teto, não ter um lugar estável para morar, se mudar mais de duas vezes em um período de seis meses, enfrentou despejo ou execução hipotecária ou teve que viver com várias famílias ou membros da família.)*	☐
**4.**	Você atualmente ou alguma vez:<br>(Assinale sim, se qualquer um for verdadeiro).<br>**a.** Ficou preocupado que a comida para seu/sua filho(a) acabasse antes de você tivesse dinheiro para comprar mais?<br>**b.** <u>OU</u> que a comida que você comprou para seu filho(a) não durou e não teve dinheiro para comprar mais?	☐
**5.**	Seu filho(a) já foi separado(a) de seus pais ou responsáveis devido a situações de acolhimento familiar ou imigração?	☐
**6.**	O seu filho(a) já morou com um dos pais/responsável que sofre de uma doença física grave ou deficiência?	☐
**7.**	Seu filho(a) já morou com um dos pais ou responsável que tenha falecido?	☐
	**Quantos “Sim” você respondeu na Parte 2?**	☐

Por favor continue respondendo o restante do questionário do outro lado da página. ⟶

PARTE 3:

Gostaríamos de entender mais sobre os pontos fortes do seu filho(a) e da sua família.

1. Quais são as melhores qualidades do seu filho(a)?

-
-
-

2. Quais são as coisas que ajudam você (ou sua família) a superar os momentos difíceis (ou lidar com o estresse)?

-
-
-
-
